# Low EGFL7 expression is associated with high lymph node spread and invasion of lymphatic vessels in colorectal cancer

**DOI:** 10.1038/s41598-023-47132-6

**Published:** 2023-11-13

**Authors:** Cristiane de Oliveira, Sandra Fátima Fernandes Martins, Paola Gyuliane Gonçalves, Gabriel Augusto Limone, Adhemar Longatto-Filho, Rui Manuel Reis, Lucas Tadeu Bidinotto

**Affiliations:** 1 Botucatu Medical School, Department of Pathology, UNESP – Univ. Estadual Paulista, Botucatu, São Paulo, Brazil; 2 Molecular Oncology Research Center, Barretos Cancer Hospital, Barretos, São Paulo, 14784 400 Brazil; 3 Life and Health Sciences Research Institute (ICVS), School of Medicine, University of Minho, Braga, Portugal; 4 ICVS/3B’s – PT Government Associate Laboratory, Braga, Guimarães, Portugal; 5 Colorectal Unit, Braga Hospital, Braga, Portugal; 6 Department of Pathology, Barretos Cancer Hospital, Barretos, São Paulo, Brazil; 7 Medical Laboratory of Medical Investigation (LIM) 14, Department of Pathology, Medical School, University of São Paulo, São Paulo, Brazil; 8 School of Medicine, University of Minho, Braga, Portugal; 9 Barretos School of Health Sciences, Dr. Paulo Prata – FACISB, Barretos, São Paulo, Brazil

**Keywords:** Gastrointestinal cancer, Tumour angiogenesis

## Abstract

Studies indicate *EGFL7* as an important gene in controlling angiogenesis and cancer growth, including in colorectal cancer (CRC). Anti-EGFL7 agents are being explored, yet without promising results. Therefore, the role of EGFL7 in CRC carcinogenesis should be investigated. This study aimed to evaluate the prognostic value of EGFL7 expression in CRC and the signaling pathways influenced by this gene. EGFL7 expression was evaluated through immunohistochemistry in 463 patients diagnosed with CRC and further associated with clinicopathological data, angiogenesis markers and survival. In silico analyzes were performed with colon adenocarcinoma data from The Cancer Genome Atlas. Analysis of enriched gene ontology and pathways were performed using the differentially expressed genes. 77.7% of patients presented low EGFL7 expression, which was associated with higher lymph node spread and invasion of lymphatic vessels, with no impact on survival. Additionally, low EGFL7 expression was associated with high VEGFR2 expression. Finally, we found in silico that *EGFL7* expression was associated with cell growth, angiogenesis, and important pathways such as VEGF, Rap-1, MAPK and PI3K/Akt. Expression of EGFL7 in tumor cells may be associated with important pathways that can alter functions related to tumor invasive processes, preventing recurrence and metastatic process.

## Introduction

Colorectal cancer (CRC) is the fourth most lethal and incident neoplasia around the world, being responsible for about 10% of the diagnostics and deaths associated with cancer^1^. The standard treatment relies in surgery, radiotherapy and/or chemotherapy, but the choice of treatment depends on disease stage, pathological characteristics, microsatellite instability status, genomic alterations, among others^2^.

About 35% of the patients present with metastatic disease, and up to 50% of those who do not present metastasis at the diagnosis will evolve with metastasis at some point of the disease^3^. In fact, angiogenesis and lymphangiogenesis are important factors in tumor maintenance and metastasization^4^. The lymphatic vessels can be influenced by tumor-derived growth factors, which can lead to lymphatic remodeling, immune function modulation, facilitating metastasization to lymph nodes and distant organs^5^. The survival, proliferation and migration of endothelial cell during the process of lymphangiogenesis depends on activation of VEGFR2/VEGFR3 receptors by VEGFC/VEGFD, stimulating the activation of protein kinase C of ERK1, ERK2 or PI3K-AKT pathways^5^.

Besides the canonical activators, studies have pointed to alternative ligands capable of modulating vessels growth. Among them, EGFL7 has been studied^6^, since it has a specific role in vascular tubulogenesis and angiogenesis regulation^7^. It has been described as an alternative Notch ligand, integrin and MAPK pathways^8^. Studies have shown that high EGFL7 expression is associated with poor prognosis in different tumor types, such as gastric cancer and CRC^9,10^.

The use of bevacizumab, an anti-VEGF monoclonal antibody^11^, combined with chemotherapy in mCRC patients showed promising results such as reduction of tumor size, increased overall survival / progression-free survival in patients with liver metastases and reduction of circulating EGFL7, associated with VEGFA reduction^11–13^. In 2013, Johnson and colleagues proposed the use of parsatuzumab, an anti-EGFL7 monoclonal antibody, for solid tumors treatment^14^. However, the phase-II studies associating chemotherapy + bevacizumab with parsatuzumab in CRC and non-small cell lung cancer did not show favorable or significant results^15,16^. Given the evidences that high EGFL7 expression led to poor prognostic events in CRC, and parsatuzumab studies did not show promisor results, the contribution of EGFL7 in CRC development, maintenance and metastization should be better investigated in order to propose more efficient treatment protocols.

Therefore, the aim of the present study is to evaluate the prognostic role of EGFL7 expression in a series of a well characterized CRC cohort and evaluate in silico the biological functions and pathways associated with differential expression of *EGFL7*.

## Material and methods

### Patients and tissue samples

Colorectal cancer samples were obtained from a well-characterized series of 463 patients who had undergone surgery at University of Minho^17^. The samples were collected from patients who underwent surgical excision of the primary tumor at Hospital of Braga (Portugal) between January of 2005 and January of 2010 and were classified by an experienced pathologist. Tumor localization was recorded and classified as colon and rectum (between anal verge and 15 cm at rigid rectoscopy). Hematoxylin and eosin staining was performed, and representative areas of the tumor were selected for tissue microarray construction. Each case was represented in the tissue microarray (TMA) by at least two cores of 0.6 mm.

### Imunohistochemistry

The 5-μm-thick sections were deparaffinized and rehydrated, and immunostaining was performed according to Brunhara et al.^18^. There was performed antigen retrieval for 20 min at 98 °C in tris–EDTA buffer and endogenous peroxidase and protein blocking were performed using Novolink Polymer Detection System (Leica Biosystems, UK). The slides were subsequently incubated with rabbit polyclonal anti-EGFL7 antibody (catalog number ab115786, Abcam, Cambridge, MA) 1:100 for 90 min at room temperature (RT). Post-primary antibody and polymer from Novolink Polymer Detection System were then placed on the slides (30 min each at RT) and chromogen color development was accomplished with 3,3’-diaminobenzidine (DAB), with a Gill-2 hematoxylin counterstain. Endothelial cells were used as an internal positive control since this antibody also labels endothelium.

The slides were blindly scored by an expert pathologist (G.A.L.) using 0 to 3 + scores^19^. The expression of EGFL7 was considered low if the score was 0–2 + ; otherwise, the expression was considered high.

The immunostaining data was associated with clinicopathological (age, gender, clinical and personal history of CRC, clinical presentation, duration of symptoms, location, CEA, presence of metastasis, tumor size, histological type, differentiation, lymph node invasion, vascular and lymphatic invasion, clinical staging and recurrence, follow-up and *status*) and immunohistochemical data of angiogenesis/lymphoangiogenesis markers—VEGFA, VEGFC, VEGFR2 and VEGFR3, retrieved from Martins et al.^17^. Finally, survival analysis was performed using Kaplan–Meier curves. Overall survival was defined as the period of analysis until death from any cause; relapse-free survival was defined as the period of analysis until any relapse detected.

The population of study was characterized by descriptive analysis, and the association between EGFL7 immunostaining and clinicopathological or angiogenesis/lymphoangiogenesis markers was performed using chi-square or Fisher’s exact test. Log rank test was performed to compare overall and relapse-free survival of patients presenting low and high EGFL7 expression. In order to analyze the impact of EGFL7 on prognosis for each tumor stage, the patients were separated by stage (1 to 4) and log rank test was performed to compare overall and relapse-free survival depending on EGFL7 expression. The results were considered statistically significant when *P* ≤ 0.05.

### In silico* analysis*

In order to analyze the molecular impact associated with *EGFL7* expression in CRC, RNA sequencing data of colon adenocarcinoma (COAD) from TCGA was analyzed. In silico analysis was performed using RTCGAToolbox^20^ and TCGAbiolinks^21^. Normalized Illumina HiSeq RSEM data from COAD was obtained, and Z-score of *EGFL7* reads was calculated for each patient. The patients were stratified in high *EGFL7* expression (above 3rd quartile, n = 109), and low expression (below 1^st^ quartile, n = 109).

The analysis of differentially expressed genes was performed using eBayes test implemented in LIMMA^22^, using as contrast the patients with high *vs.* low *EGFL7* expression. The genes presenting False Discovery Rate (FDR) ≤ 0.05 and Fold Change ≥|2.0| were considered differentially expressed.

The list of differentially expressed genes was submitted to Enriched Gene Ontology (GO) gene set enrichment analyses and Kyoto Encyclopedia of Genes and Genomes (KEGG)^23,24^ enrichment analyses using clusterProfiler^25^. Similarity of the terms was determined using enrichplot package implemented in R. The GO and KEGG terms were considered statistically significant when FDR ≤ 0.05.

### Ethical approval

This study protocol was reviewed and approved by the Ethics Committee of University of Minho (number 32/2013) and Barretos Cancer Hospital (number 1955/2020). This study was performed in line with the principles of the Declaration of Helsinki.

### Consent participate

Written informed consent was obtained from participants to participate in the study.

## Results

### Imunohistochemistry

**Figure 1:**
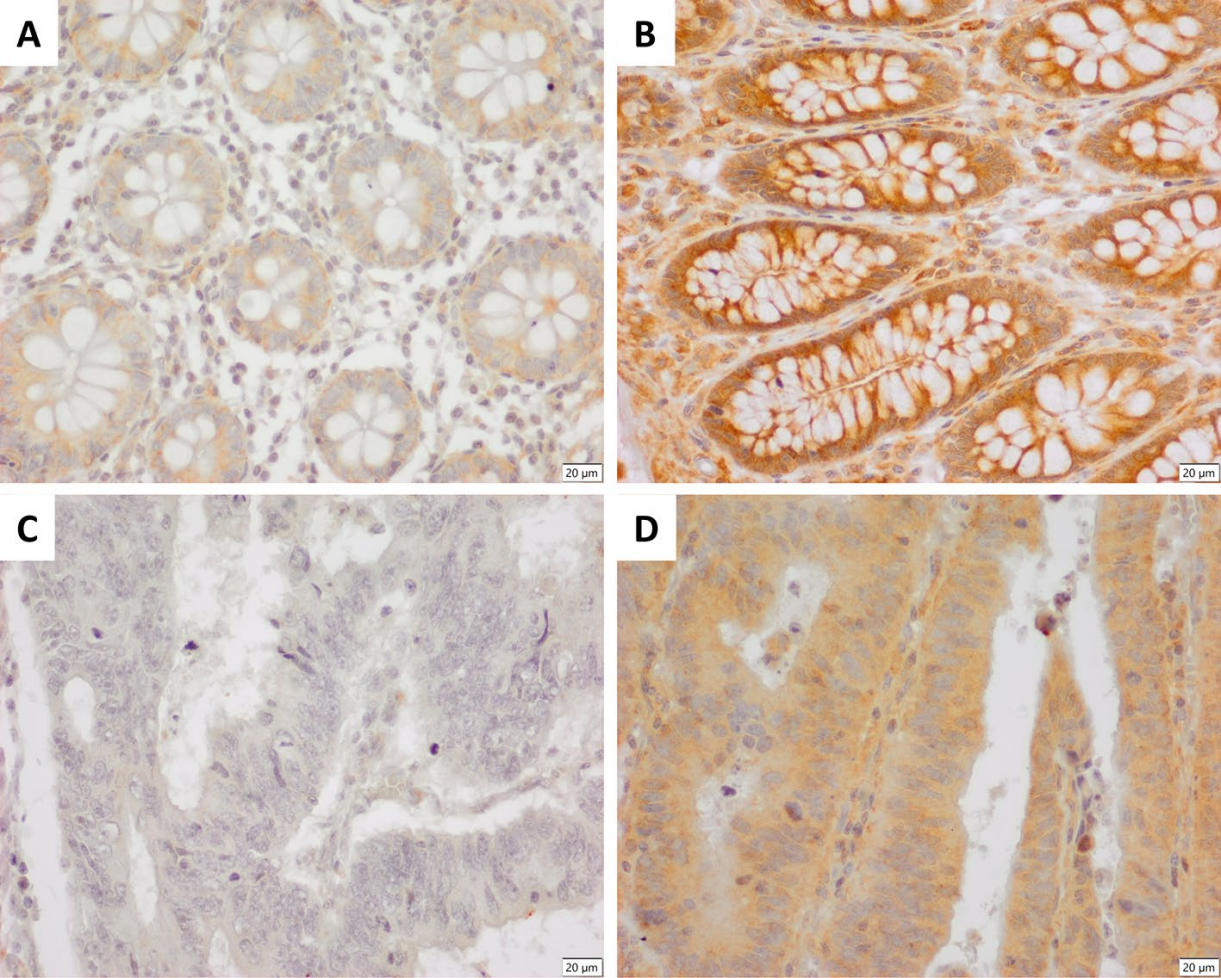
Representative immunohistochemical expression of EGFL7 in normal colorectal tissue (**A** and **B**) and colon adenocarcinoma (**C** and **D**). **A**. enterocytes weakly cytoplasmatic positive (+/3+); **B**. enterocytes strongly positive (+++/3+) with minor background on stromal cells; **C**. adenocarcinoma with negative expression; **D**. adenocarcinoma mildly citoplasmatic positive (++/3+). Scale bars of 20 micrometers.

We found high expression of EGFL7 protein by immunohistochemistry in 103 out of 463 patients (22.3%). The staining was essentially cytoplasmatic in all cases (shown in Fig. 1). There was found no association of EGFL7 expression and clinopatiological features (Table 1). The analysis of EGFL7 expression and pathological data showed that low EGFL7 expression was associated with presence of spread to lymph nodes and lymphatic vessel invasion (*P* < 0.05, Table 1). Moreover, there was found association between low EGFL7 expression and the expression of VEGFR2 (*P* < 0.05, Table 2).

**Table 1:** Assessment of association of EGFL7 expression with clinicopathological features.

Clinicopathological features		EGFL7 expression		*P* value
		Low n (%)	High n (%)	
Sex	Male	227 (78.5%)	62 (21.5%)	0.597
	Female	133 (76.4%)	41 (23.6%)	
Age (years)	< = 45	16 (72.7%)	6 (27.3%)	0.36
	> 45	344 (78.0%)	97 (22.0%)	
Location	Ascending colon	90 (81.8%)	20 (18.2%)	0.502
	Descending colon	176 (76.5%)	54 (23.5%)	
	Rectum	94 (76.4%)	29 (23.6%)	
CEA (ng/mL)	< = 10	243 (76.2%)	76 (23.8%)	0.207
	> 10	59 (83.1%)	12 (16.9%)	
Tumor size (cm)	< = 4.5	207 (76.7%)	63 (23.3%)	0.398
	> 4.5	133 (80.1%)	33 (19.9%)	
Histological type	Adenocarcinoma	320 (77.1%)	95 (22.9%)	0.061
	Mucinous	39 (86.7%)	6 (13.3%)	
	Signet ring & mucinous	1 (33.3%)	2 (66.7%)	
Differentiation	Well	151 (75.1%)	50 (24.9%)	0.398
	Moderate	157 (78.5%)	43 (21.5%)	
	Poor	38 (86.4%)	6 (13.6%)	
	Undifferentiated	2 (66.7%)	1 (33.3%)	
Spread to lymph nodes	Absent	193 (73.7%)	69 (26.3%)	0.020*
	Present	156 (83.0%)	32 (17.0%)	
Vessel invasion	Absent	197 (79.1%)	52 (20.9%)	0.579
	Present	150 (76.9%)	45 (23.1%)	
Lymphatic invasion	Absent	142 (73.6%)	51 (26.4%)	0.033*
	Present	197 (82.1%)	43 (17.9%)	
TNM stage	1	55 (70.5%)	23 (29.5%)	0.218
	2	134 (77.0%)	40 (23.0%)	
	3	116 (79.5%)	30 (20.5%)	
	4	55 (84.6%)	10 (15.4%)	
Relapse	Absent	206 (77.7%)	59 (22.3%)	0.505
	Present	82 (74.5%)	28 (25.5%)	

*Statistically significant (*p* ≤ 0.05).

**Table 2:** Assessment of association of EGFL7 expression with angiogenesis biomarkers (Data obtained from^17^).

Molecular markers		EGFL7 expression		*P* value
		Low n (%)	High n (%)	
VEGFA expression	Negative	8 (88.9%)	1 (11.1%)	0.691
	Positive	346 (77.4%)	101 (22.6%)	
VEGFC expression	Negative	31 (86.1%)	5 (13.9%)	0.210
	Positive	326 (77.1%)	97 (22.9%)	
VEGFR2 expression	Negative	7 (53.8%)	6 (46.2%)	0.049*
	Positive	344 (78.2%)	96 (21.8%)	
VEGFR3 expression	Negative	271 (79.0%)	72 (21.0%)	0.219
	Positive	83 (73.5%)	30 (26.5%)	

*Statistically significant (*p* ≤ 0.05).

**Figure 2:**
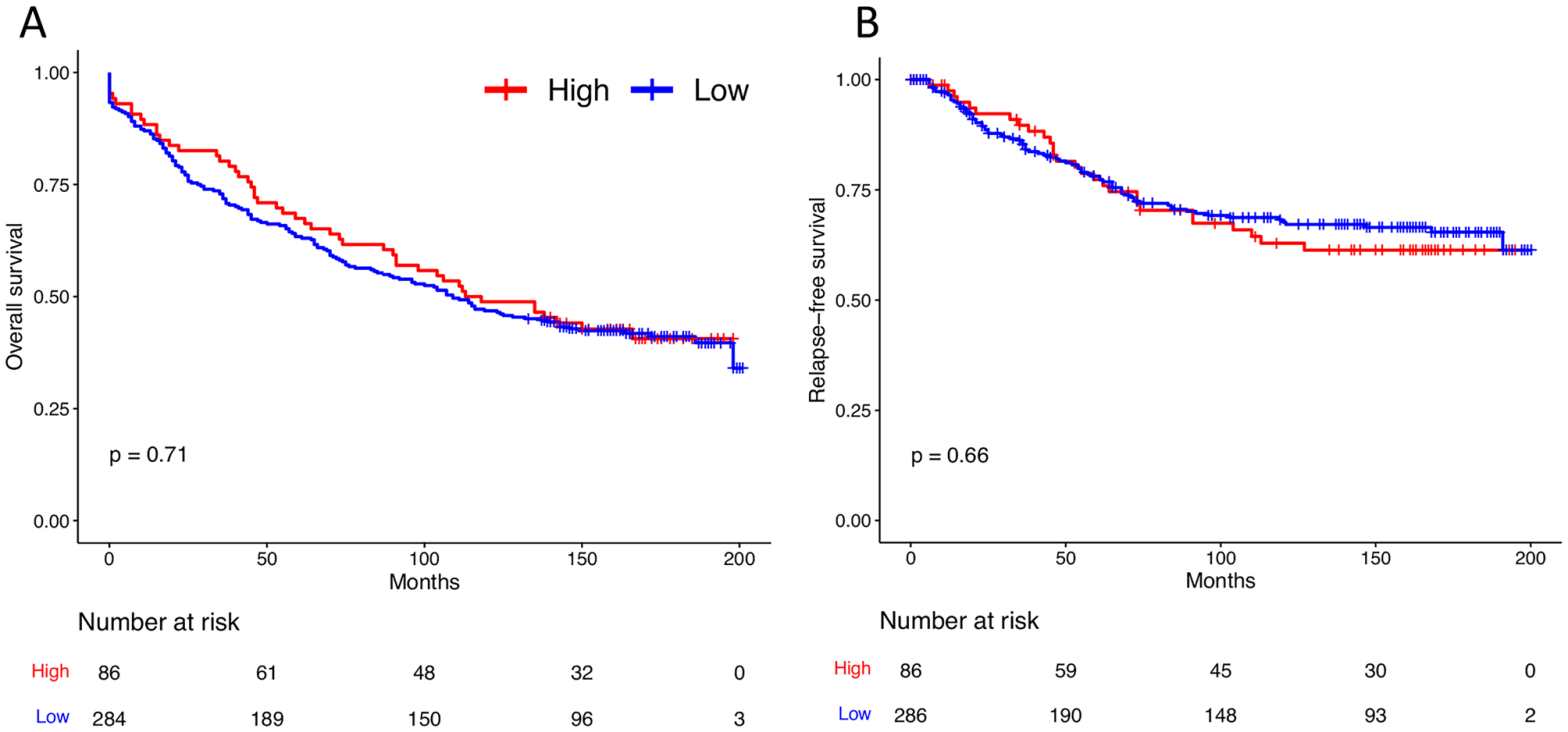
Overall- (**A**) and recurrence-free (**B**) survival of colorectal patients considering EGFL7 immunolabeling.

Overall and recurrence-free survival was not different between low and high expression of EGFL7 in colorectal cancer (shown in Fig. 2). The overall survival (OS) of 50% of the patients was close to 110 months. Regarding tumor localization (ascending colon, descending colon, or rectum, shown in Supplementary Fig. 1), there was no difference in OS and RFS in the comparison of patients with low and high expression of EGFL7. Finally, although obvious differences in survival can be noted among tumor stages (stages 1, 2, 3 or 4, shown in Supplementary Fig. 2), the comparison of the curves (low EGFL7 expression vs. high EGFL7 expression) within each stage (stages 1, 2, 3 or 4) was not statistically different (*P* > 0.05).

### In silico* analysis*

In order to characterize the pathways and biological processes associated with *EGFL7* expression, we performed in silico analysis using RNA sequencing data from The Cancer Genome Atlas (TCGA). Differential expression of patients presenting high *EGFL7* expression vs. low *EGFL7* expression presented 1,718 differentially expressed genes, being 103 downregulated and 1,615 upregulated (Supplementary Table 1).

**Figure 3:**
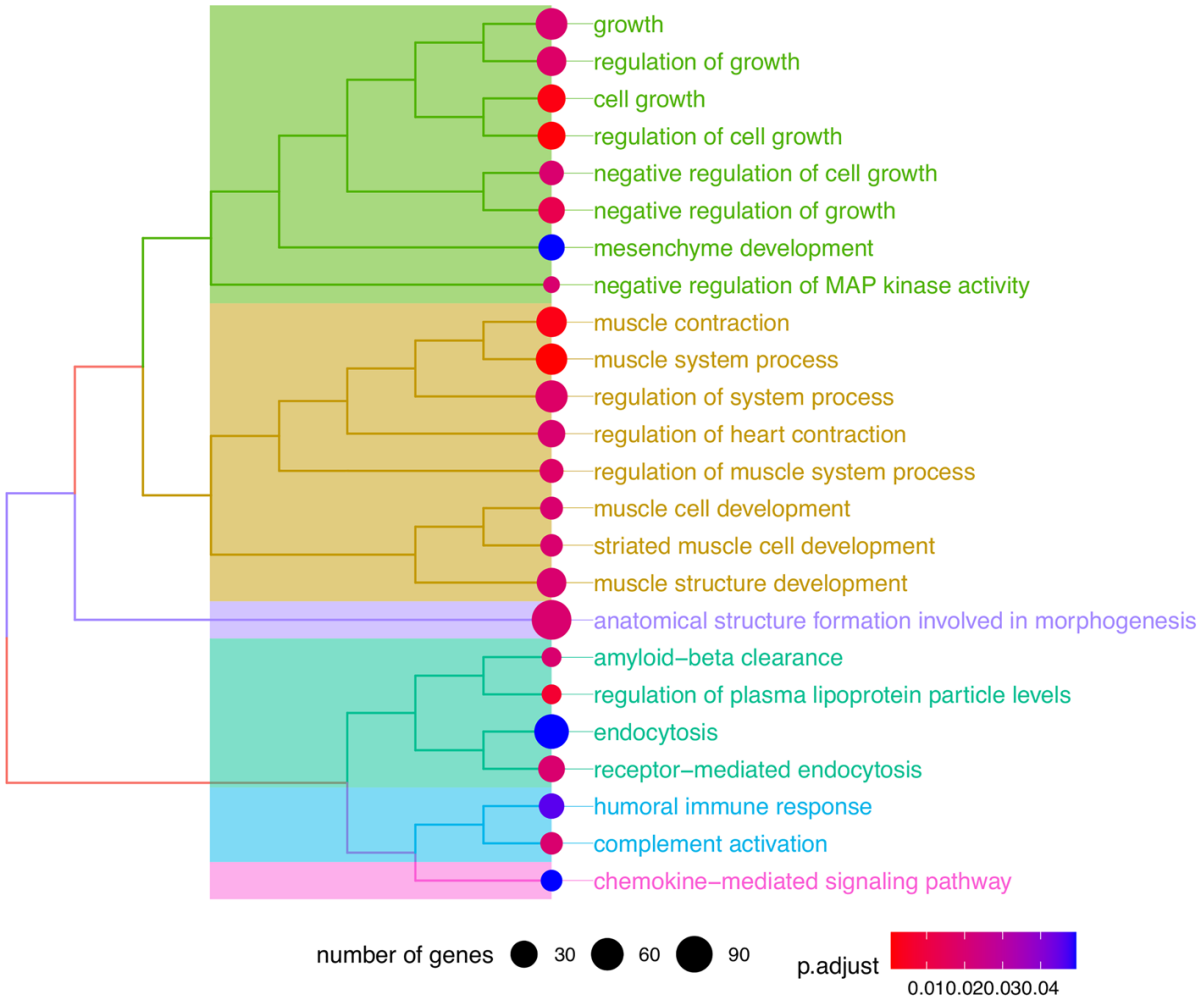
Gene ontology terms enriched in the differentially expressed genes of colon adenocarcinoma patients (The Cancer Genome Atlas) with high EGFL7 expression compared with low EGFL7 expression.

Enriched gene ontology (GO) gene set enrichment analysis revealed 24 enriched terms. Of note, 8 terms (shown in Fig. 3) were aggregated under a cluster associated with growth/development. The genes present in the GO terms are found in Supplementary Table 2.

**Figure 4:**
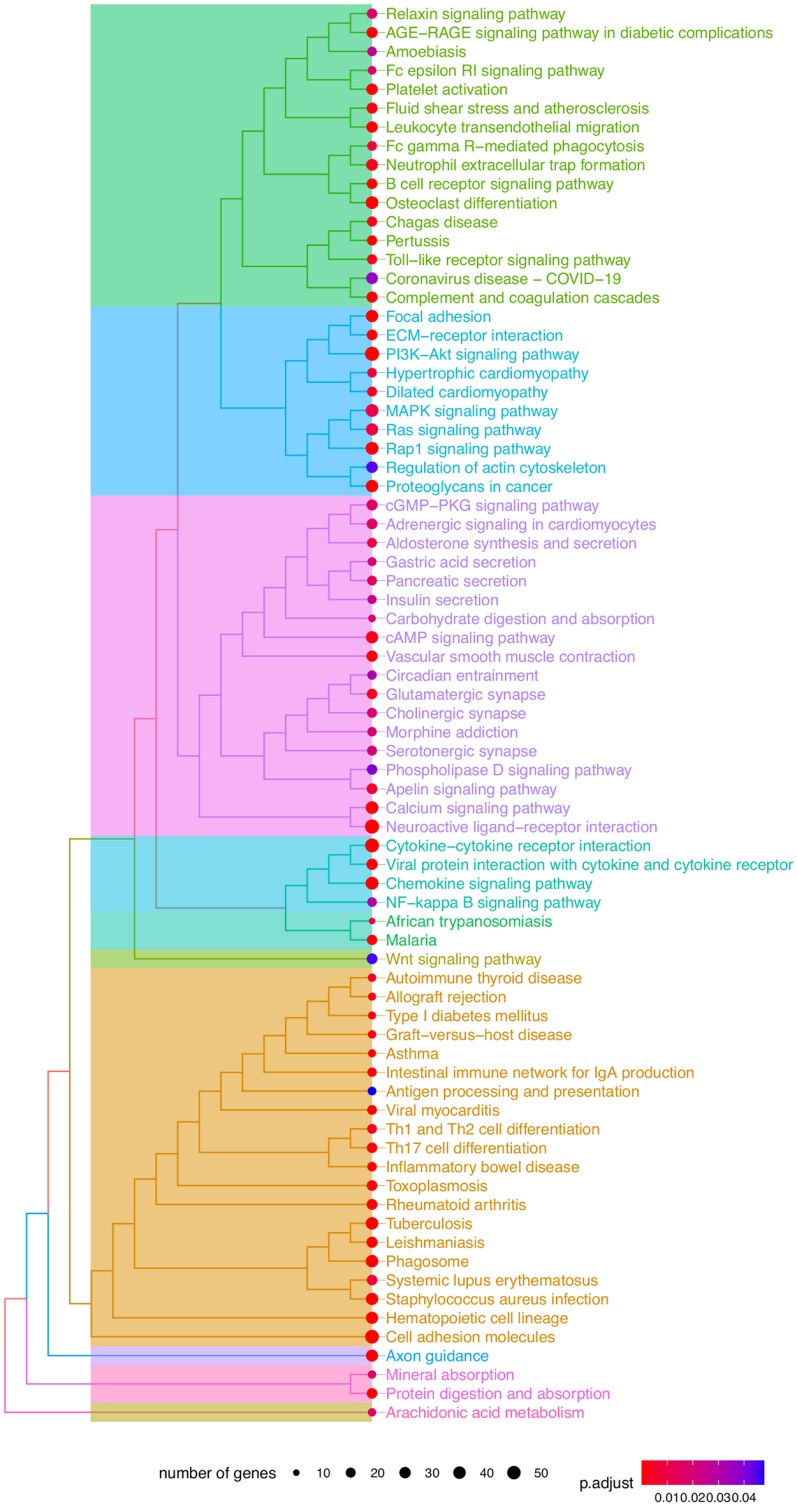
KEGG (Kyoto Encyclopedia of Genes and Genomes) terms enriched in the differentially expressed genes of colon adenocarcinoma patients (The Cancer Genome Atlas) with high EGFL7 expression compared with low EGFL7 expression.

Enriched KEGG analysis revealed 75 enriched pathways. Of note, we found a cluster which include several pathways associated with cancer development (Focal adhesion, ECM-receptor interaction, PI3K-Akt signaling pathway, MAPK signaling pathway, Ras signaling pathway, RAP1 signaling pathway and Proteoglycans in cancer) (shown in Fig. 4). The genes present in the KEGG terms are found in Supplementary Table 2. Of note, Wnt signaling pathway was also enriched in our analysis (shown in Fig. 4, Supplementary Table 2).

## Discussion

The present study aimed to evaluate the prognostic potential of EGFL7 expression in patients diagnosed with CRC and to propose biological processes and pathways altered by the differential expression of this gene. We found that the low expression of EGFL7 in neoplastic cells was associated with greater lymph node involvement and lymphatic vessel invasion, possibly due to dysregulation of carcinogenesis-related processes (cell growth, cell adhesion, angiogenesis) through important pathways such as proteoglycans in cancer, Ras, Rap1, MAPK and PI3K/Akt.

There are several studies regarding EGFL7 expression in different tumor types. The high expression of this gene was found as a marker of worse prognosis in several tumor types^9,10,26^. In addition, the interaction of EGFL7 and EGFR leads to the activation of important downstream pathways related to the development and growth of several types of tumors. High expression of EGFL7 in gliomas^26^ and in metastatic gastric cancer^27^ promotes the activation of the AKT and ERK pathways through the interaction in EGFR; in hepatocellular cancer^28^ EGFL7 promotes metastasis through activating FAK phosphorylation by binding EGFR. Additionally, in renal cancer^29^, the activation of the EGFL7/EGFR/FAK pathway induces the migration of endothelial cells, inducing the formation of vascular tubes contributing to tumor progression. Overall, the EGFL7/EGFR signaling pathway may play an important role in intratumoral angiogenesis, metastasis and invasion^8^. We previously observed that high EGFL7 expression was associated with worse clinical outcome in patients diagnosed with pilocytic astrocytoma^18^, and worse survival and lower Karnofsky Performance Score in glioblastoma^30^.

Hansen et al. analyzed the expression of EGFL7 to estimate vessel area in mCRC, and found positive association with *KRAS* mutation^31^. Although the mechanism is still poorly characterized, possibly the increase in MAPK pathway can upregulate the expression of *EGFL7*, leading to an increase in (lymph)angiogenesis and tumor aggressiveness^32^. Subsequently, they described that the intratumoral endothelial expression of this protein is higher in primary tumors of patients diagnosed with stage II or III CRC who had recurrence^33^. Finally, this group described reduction of circulating EGFL7 of mCRC patients after chemotherapy^13^. In addition, a worse prognosis was found in those who had high amounts of baseline cir-EGFL7 before treatment^13^. In patients with liver metastases who underwent bevacizumab-based chemotherapy followed by surgical resection, low intratumoral expression of *EGFL7* mRNA in metastases was associated with higher disease-free survival^34^. In addition to these encouraging data, the study by Hansen et al.^33^ suggests an important predictive value of EGFL7-positive vascular area in relation to first-line chemotherapy and bevacizumab for CRC and suggests the use of a dual VEGFA-EGFL7 blocking mechanism. In contrast, our data suggest that EGFL7 expression in tumor parenchyma is not associated with differences in overall survival and, additionally, low expression is associated with increased lymph node spread and invasion of lymphatic vessels of metastatic colorectal cancer. We suggest that the literature data found analyzing only EGFL7 expression in vessels^13,31,33^ may be more associated with angiogenesis per se than to the effects of EGFL7 expression.

This evidence can be indirectly supported by ongoing studies, such as Garcia-Carbonero and colleagues^15^, who showed that treatment with parsatuzumab (anti-EGFL7 antibody that selectively blocks the interaction of EGFL7 and endothelial cells) failed to improve the efficacy of FOLFOX + bevacizumab combination in patients with mCRC. Similarly, another phase II randomized clinical trial^16^ showed that administration of parsatuzumab to non-small cell lung cancer patients did not improve treatment (bevacizumab + carboplatin/placlitaxel) efficacy; still, patients who received parsatuzumab had lower progression-free survival than placebo arm. Therefore, the effect of blocking EGFL7 expression by parsatuzumab led to exiguous results compared to blocking VEGF, suggesting that the main effect of this combination was associated with angiogenesis and not to EGFL7 expression.

Our in silico data show enriched biological processes and pathways related to growth, mesenchyme development, regulation of PI3K/Akt, MAPK, Wnt signaling and Rap1 pathway, thus strengthening the potential molecular mechanism of EGFL7 in mediating CRC^35^. Activation of the PI3K/Akt pathway together with mTOR can regulate several biological processes important for growth, metabolism, autophagy, and angiogenesis^36^. This pathway regulates angiogenesis by increasing VEGF secretion, modulating the expression of NO and angiopoietins^36,37^. The binding of VEGF to receptors on endothelial cells stimulates the activation of this pathway, which is essential for endothelial cell migration, being fundamental for the development of blood vessels^37^. VEGFR2 activation increases signaling of several pathways, such as MAPK and PI3K/Akt/mTOR^38^, found altered in our in silico analysis. Altogether, the positive expression of VEGFR2 may have increased lymphangiogenesis in patients with low EGFL7 expression. This, therefore, led to greater lymph node spread, since the lymphatic pathway is the main route of spreading of neoplastic cells in CRC^6^. Importantly, in CRC, the involvement of lymph node and lymphatic invasion are important factors to be considered when determining treatment^6^.

In addition to the dysregulation of the widely studied pathways in carcinogenesis cited above, our in silico analysis also found other less studied pathways and biological processes, such as the Rap1. RAP1 has potential to regulate and mediate Ras functions, as well as being related to many of the characteristics of cancer^39^, acting as a central regulator of adhesion, motility cellularity, cell polarity, and migration^39^. Furthermore, RAP1 promotes vascular endothelial growth factor receptor 2 (VEGFR2) activation and angiogenesis through integrins. Thus, RAP1 plays an important role in invasion and metastasis due to its regulation of cell adhesion and cytoskeletal remodeling through ERK/MAPK signaling and integrin activation^40^. There is evidence that RAP1 activation promotes tumorigenesis in several systems^39^. In CRC, activation of RAP1 resulted in impaired cell adhesion and increased cell–matrix adhesion, inducing the spread of neoplastic cells. Therefore, activation of RAP1 is associated with several biological processes such as cellular metabolism, cytoskeletal remodeling, cell proliferation, migration and metastasis through the regulation of downstream pathways such as ERK, AKT, FAK and Wnt^36,40^.

We conclude, therefore, that the low expression of EGFL7 in the tumor cells of patients diagnosed with CRC may be associated with high expression of VEGF2, thus leading to an increase in lymphatic invasion and greater lymphangiogenesis. Our in silico analysis indicates that EGFL7 expression is associated with important pathways related to carcinogenesis and lymphangiogenesis. Further studies are needed to validate the findings identified in silico, and to lighten the association of these results with clinicopathological findings to elucidate the mechanism of EGFL7 in the genesis of CRC, in order to propose adequate treatment approaches for colorectal cancer using EGFL7 as possible biomarker.

### Supplementary Information

Supplementary Figure 1.

Supplementary Figure 2.

Supplementary Table 1.

Supplementary Table 2.

## Data Availability

The datasets analyzed during the current study are available in the TCGA repository, [https://www.cancer.gov/tcga].
