# Supplementary figures and images for: Low EGFL7 expression is associated with high lymph node spread and invasion of lymphatic vessels in colorectal cancer
Source: Sci Rep. 2023 Nov 13;13:19783. doi: 10.1038/s41598-023-47132-6 (PMC10643678; doi:10.1038/s41598-023-47132-6)

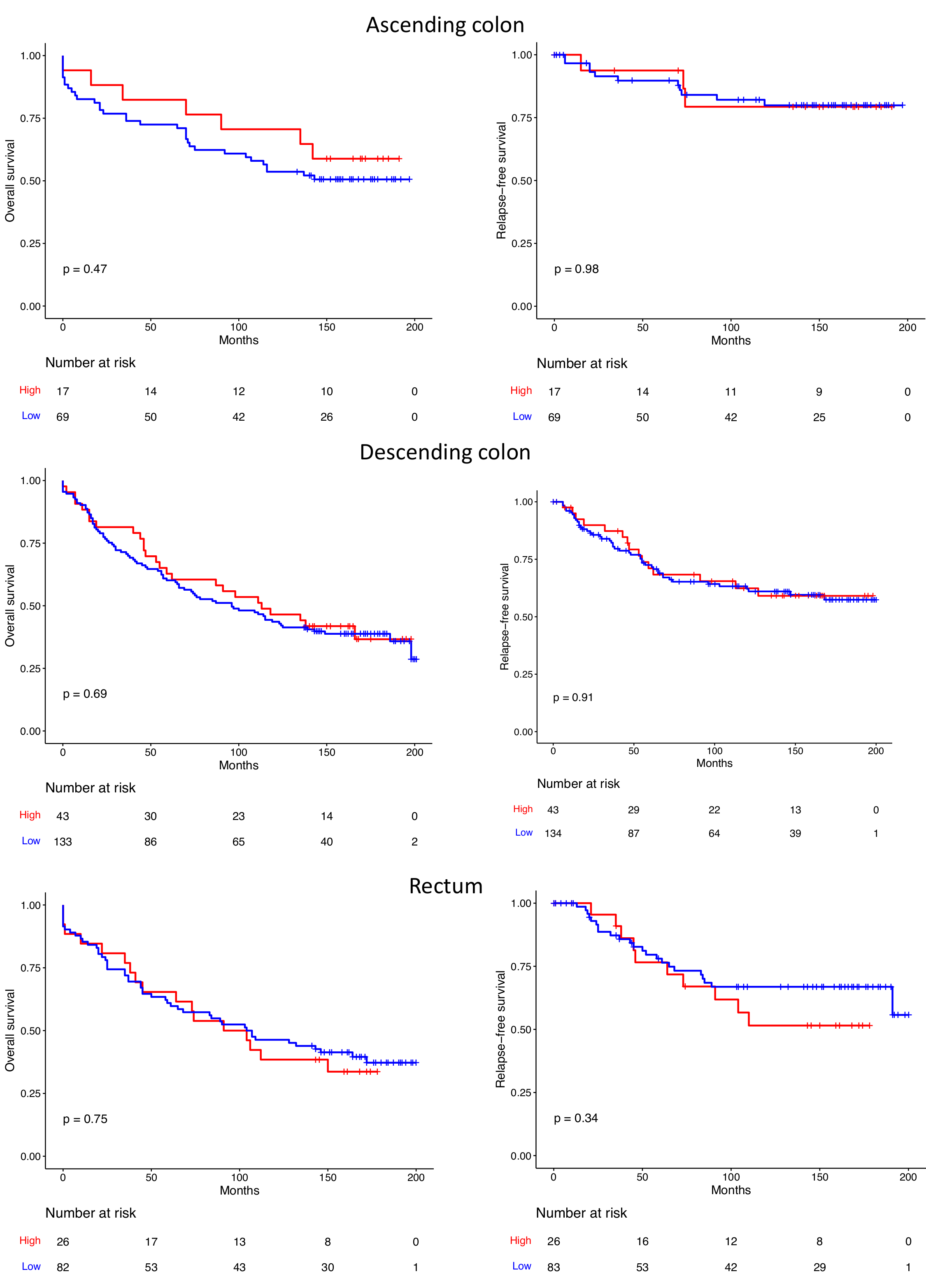

Supplement: Supplementary file 1 — Supplementary Figure 1. [file 41598_2023_47132_MOESM1_ESM.tiff]

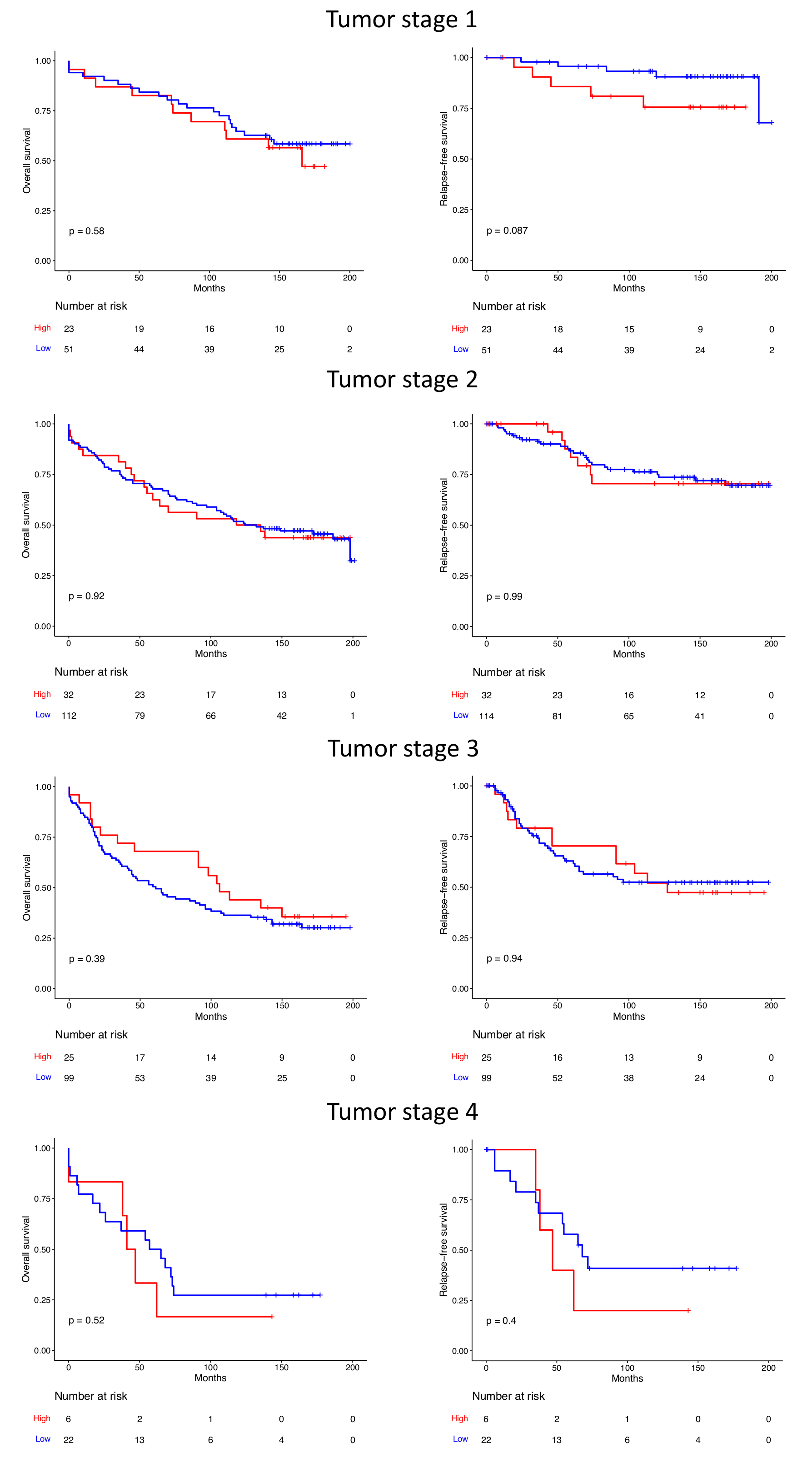

Supplement: Supplementary file 2 — Supplementary Figure 2. [file 41598_2023_47132_MOESM2_ESM.tiff]
